# Many Honours for Hospital Workers

**Published:** 1917-09-01

**Authors:** 


### THE TWO NEW ORDERS.

### Many Honours for Hospital Workers.

THE publication of the first list of appointments to the two new Orders, the Order of the British Empire and the Order of the Companions of Honour, which have been instituted in recognition of the various war services rendered by British subjects and their Allies, has a special interest.

THE ORDER OF THE BRITISH EMPIRE has five classes for men and five for women; members of the first two classes in either division have the right to prefix the title "Sir" or "Dame" to their names. It is very satisfactory to note that, almost without exception, this, the first list of recipients of the new Orders, is, on the whole, freer from grounds of criticism than any non-warriors list which has appeared in the *London Gazette* for years. It is only just that this tribute should be paid to those who have been charged with the exceedingly difficult responsibility of preparing the list, and to His Majesty the King, with whom the final revision in this case presumably rested. Among the 268 appointments the hospital world will note with interest the following names:—

### The Knights Grand Cross (G.B.E.)

include the Hon. Arthur Stanley, C.B., C.V.O., M.P., Chairman of the British Red Cross Society, and Sir Arthur Pearson, Bart., Chairman of the Blinded Sailors' and Soldiers' Care Committee.

### The Dames Grand Cross (G.B.E.), of which the Queen is the first member, include Lady Wemyss Paget, Serbian Relief Fund, and the Hon. Lady Lawley, Hon. Sec. of Queen Mary's Needlework Guild.

### The Knights Commanders (K.B.E.)

include Sir Edward Cook, Joint Director of the Press Bureau and the Biographer of Florence Nightingale; the Hon. W. H. Goschen, Chairman King George V. Hospital and Deputy-Chairman of the Joint Finance Committee Red Cross and Order of St. John of Jerusalem; Hon. Lieut.-Col. Edward Stewart, Medical Assessor to the Red Cross and Society of St. John of Jerusalem.

### The C.B.E. (Commanders)

is given to Dr. Garrett Anderson, who organised the first hospital to be managed by women at the Front; Mrs. Cecil Baker, Hon. Sec. British Ambulance Committee; Lady Florence Elizabeth Barrett, for her work for the British Red Cross Society; Miss Gertrude Bell, for her special war work in the East and her services to the Red Cross; Mrs. Helen May Gaskell, Hon. Sec. British Red Cross Society and the Order of St. John of Jerusalem War Library; Lieut.-Col. Jay Gould, M.B., I.M.S., late Commissioner British Red Cross Society, Mesopotamia; Thomas Wheeler Hart, Esq., M.B., County Director British Red Cross Society, East Lancashire; Miss Margaret Hogg, Matron Guy's Hospital; Miss Eva Lückes, Matron London Hospital; Miss Annie McIntosh, Matron St. Bartholomew's Hospital; Dr. Flora Murray, Doctor in Charge Endell Street Hospital; the Hon. Lady Norman, who took a hospital to France in 1914, and for her special services to the British hospitals there has been mentioned in the Commander-in-Chief's dispatches; Sister Pauline, Sister of Charity Italian Hospital, Queen Square; Dr. Mary Scharlieb; Lady Sophie Beatrix Mary Scott, Head of the Gifts Department Stores British Red Cross Society; Miss Alicia Lloyd Still, Matron St. Thomas's Hospital and Superintendent Nightingale Training School; Capt. M. F. Colchester-Wemyss, County Director British Red Cross Society, Gloucestershire.

### The Officers (O.B.E.)

include Wilfred Balgarnie, Esq., M.B., Assistant County Director British Red Cross Society, Hampshire; D. R. P. Beresford, Esq., Hon. Sec. County of Dublin Branch British Red Cross Society; Miss Ethel Birkin, Matron British Red Cross Auxiliary Hospital, Nottingham; Capt. F. B. Langridge, Assistant Secretary to the Commissioners British Red Cross Society, Boulogne; D. M. Millar, Esq., Medical Services British Red Cross Society, Durham; Henry Musgrave, Esq., for his work in connection with Hospital War Funds; W. E. Nelson, Esq., Assistant County Director British Red Cross Society, Warwickshire; Lady Muriel Paget, who raised and organised the Anglo-Russian Hospital; Miss Elsie Saunders, Organiser and Secretary British Red Cross Society, Derbyshire; Mrs. Lena Simpson (Miss Lena Ashwell), Organiser of Entertainments for the Troops, whose concerts in the hospitals in France have been described more than once in THE HOSPITAL.

### The Members (M.B.E.)

include Miss R. M. Ard, Commandant Rusthall V.A.D. Hospital, Tunbridge Wells; Sub.-Lieut. E. S. Beard, R.N.V.R., Quartermaster and Secretary Royal Naval Auxiliary Hospital, Truro; S. J. Chesterton, Esq., Section Leader British Red Cross Company, Italy; Mrs. J. H. Fisher, Commandant Balgowan Hospital, Beckenham; G. C. Franklin, Esq., F.R.C.S., LL.D., Medical Officer in Charge Hawkstone Red Cross Hospital, Farnham; Miss E. P. Hughes, late Commandant and Organiser British Red Cross Society, South Wales; Mrs. C. A. Hunter, Quartermaster Hoole's House Hospital, Chester; Miss K. A. B. Landon, Assistant Commandant Devon V.A.D. 66 and Exmouth Hospital; George Price, Esq., Storekeeper Headquarters, British Red Cross Society, Malta; L. C. Tricker, Esq., Storekeeper British Red Cross Society, Malta; Harold Wilkins, Esq., Head of Passports Department, British Red Cross Society.

(Continued on page 442.)

### THE TWO NEW ORDERS (*continued from page 443*).

THE ORDER OF THE COMPANIONS OF HONOUR.

The Marchioness of Lansdowne, Member of the Council of the Red Cross, and Miss Elizabeth Haldane, Vice-Chairman Advisory Council of the Territorial Force Nursing Association, are included in this Order, which carries no title or precedence, and is conferred on a limited number of people, as a special honour dissociated both from the acceptance of title and the classification of merit. Only seventeen appointments to it have so far been made.

The Statutes of the new Order designated it as "The Most Excellent Order of the British Empire," and are published in a Supplement to the *London Gazette* of August 24, 1917. The *Times* of August 25, page 8, column 4, gives full extracts from the ordinations.

